# Effects of Habitat Fragmentation on Abundance, Larval Food and Parasitism of a Spider-Hunting Wasp

**DOI:** 10.1371/journal.pone.0059286

**Published:** 2013-03-14

**Authors:** Valérie Coudrain, Felix Herzog, Martin H. Entling

**Affiliations:** 1 Research Station Agroscope Reckenholz-Tänikon ART, Zürich, Switzerland; 2 Institute of Ecology and Evolution, University of Bern, Bern, Switzerland; 3 Institute for Environmental Sciences, University of Koblenz-Landau, Landau/Pfalz, Germany; Consiglio Nazionale delle Ricerche (CNR), Italy

## Abstract

Habitat fragmentation strongly affects species distribution and abundance. However, mechanisms underlying fragmentation effects often remain unresolved. Potential mechanisms are (1) reduced dispersal of a species or (2) altered species interactions in fragmented landscapes. We studied if abundance of the spider-hunting and cavity-nesting wasp *Trypoxylon figulus* Linnaeus (Hymenoptera: Crabronidae) is affected by fragmentation, and then tested for any effect of larval food (bottom up regulation) and parasitism (top down regulation). Trap nests of *T. figulus* were studied in 30 agricultural landscapes of the Swiss Plateau. The sites varied in the level of isolation from forest (adjacent, in the open landscape but connected, isolated) and in the amount of woody habitat (from 4 % to 74 %). We recorded wasp abundance (number of occupied reed tubes), determined parasitism of brood cells and analysed the diversity and abundance of spiders that were deposited as larval food. Abundances of *T. figulus* were negatively related to forest cover in the landscape. In addition, *T. figulus* abundances were highest at forest edges, reduced by 33.1% in connected sites and by 79.4% in isolated sites. The mean number of spiders per brood cell was lowest in isolated sites. Nevertheless, structural equation modelling revealed that this did not directly determine wasp abundance. Parasitism was neither related to the amount of woody habitat nor to isolation and did not change with host density. Therefore, our study showed that the abundance of *T. figulus* cannot be fully explained by the studied trophic interactions. Further factors, such as dispersal and habitat preference, seem to play a role in the population dynamics of this widespread secondary carnivore in agricultural landscapes.

## Introduction

Habitat fragmentation can strongly affect species distribution and abundance with consequences for ecosystem functioning and related ecosystem services [1], [2], [3], [4]. Fragmentation studies need to distinguish between (i) habitat loss, which is the reduction in total amount of habitat within the landscape, and (ii) fragmentation *per se*, which is the breakdown of habitat into smaller patches that become isolated from one another [5]. These two processes can have different effects on the abundance and diversity of organisms [6], [7], [8], [9], [10]. Whereas this issue is increasingly being recognised, the mechanisms underlying species response to fragmentation often remain unresolved. This information is, however, essential to maintain or restore effective landscape connectivity [11], [12], [13], [14]. The effects of fragmentation can result from (1) altered migration of a species in fragmented habitats, caused e.g. by limited dispersal ability of the species [15], [16], or by the hostility of the surrounding matrix and reduced availability of shelter and nesting opportunities [17], [18], [19]. Alternatively, it can result from (2) altered species interactions in fragmented habitats, such as reduced food availability [20], [21], [22], or increased parasitism [23], [24].

The rarity of studies that distinguish between different mechanisms behind fragmentation effects may be partly due to the difficulty to quantify trophic interactions in the field.

Quantifying food availability to a species is difficult owing to factors, such as fluctuating spatial and temporal prey abundance and the ability of the species to access to the resources.

Studying the body condition of a species in different landscapes can indicate if fragmentation effects are due to altered food availability [22], [25]. More direct evidence for fragmentation effects on food availability can be revealed by studying the actual diet of a species. Here, trap-nesting *Trypoxylon* wasps offer an ideal study system because prey items deposited as larval food can be collected and determined to species level [26], [27], [28], [29].

Moreover, trap-nests allow a reliable assessment of parasitism rate of brood cells.

Habitat loss and fragmentation can destabilize host-parasitoid relationships [30], [31]. According to the trophic levels hypothesis, higher trophic levels, such as parasitoids, should suffer more from fragmentation than their hosts [32], [33]. As a consequence, hosts may be released from their parasitoids in isolated habitats [26], [30], [34]. However, this hypothesis has been challenged [35], [36] and the number of studies investigating parasitism in relation to habitat loss and isolation is still limited [37].

We studied the impact of fragmentation on the mud-dauber wasp *Trypoxylon figulus* Linnaeus (Hymenoptera: Crabronidae). This wasp nests in cavities in wood or other plant material and readily colonizes artificial trap-nests. It hunts spiders as larval food [38], [39], [40]. Diet and brood parasitism of *T. figulus* were studied in 30 trap-nest locations that varied (a) in their isolation from other woody habitats and (b) in the percentage of woody habitats in the surrounding landscape. We predicted that (i) the abundance of *T. figulus* decreases with increasing habitat isolation and / or decreasing amount of woody habitat. We expected (ii) higher number and/or mean weight of spiders per brood cell in sites with higher abundance of *T. figulus*. We further expected that (iii) if *T. figulus* hunts spiders according to their abundance in the landscape, its diet will vary as a function of the surrounding landscape, with higher frequencies of forest-related spider species in habitats adjacent to forest and/or landscapes with large amounts of woody habitats. Eventually, according to the trophic-level hypothesis, we predicted lower parasitism rate in isolated sites (iv).

## Materials and Methods

### Ethics statements

All sites were visited with landowner permission, and no permits for sampling were required. Our study did not involve any endangered or protected species.

### Study sites

We conducted our study in agricultural landscapes of the Swiss Plateau between the cities of Bern, Solothurn and Fribourg. Thirty experimental sites were chosen over an area of 23×32 km varying in altitude between 465 and 705 m above sea level [4]. Each site consisted of an 18-m-long row of seven 6-year-old cherry trees that had been planted on permanent grassland in spring 2008 and since then managed in a standardized manner [26]. Sites were selected according to the amount of woody habitat (forest, hedges, orchards and single trees) within a 500 m radius (range from 4 to 74 %) and their isolation from woody habitat: ten sites were located at the edge of dense, tall growing forest (adjacent), twenty sites were located at a distance of 100–200 m from the next forest, half of them connected by small-sized woody structures such as hedgerows or single trees (connected) and the other half isolated from any woody habitat (isolated). 500 m radius was chosen based on the maximal foraging range of solitary bees of similar body size [41]. Information on woody habitats was derived from official digital land-use maps (vector25, swisstopo, Wabern) and verified using aerial photographs and field inspection. There was no statistical dependency between the percentage of woody habitat cover and the level of isolation (F_2,27_  =  0.004, *P*  =  0.99).

### Trap nests

Since 2008, four trap nests for solitary bees and wasps were placed in each site. Trap-nests consisted of plastic cylinders filled with on average 180 tubes of common reed (*Phragmites australis* L.) of diameter 2–10 mm (same proportion in each nest). Reed tubes will be referred hereafter as nesting tubes. Two of the four trap nests had been removed each year in October to analyse wasp and bee communities [26], and two had been left in the field to develop a local population over 3 consecutive years. For this study, two trap nests per site were monitored fortnightly from April to July 2010. Nesting tubes closed with mud, indicating a completed nest, were collected and brought to the laboratory. A short time interval between each tube collection was important to obtain the spider prey before it was eaten by the developing wasp larvae. The nesting tubes were carefully opened to extract the prey content of two cells with no spiders eaten yet. As the laying sequence is often non-random in solitary Hymenoptera, with female eggs being laid first and usually provisioned more [42], [43], [44], we selected one cell located at the bottom of the reed tube and the other near the entrance. The tubes were closed again so that the wasp larvae in the remaining cells could develop for subsequent species identification. The number of spiders per cell was counted and the spiders were determined as close as possible to the species level. The fresh weight of the total spider content of 202 cells with undamaged spider individuals was measured. Total spider weight per cell and the number of spiders per cell were positively correlated (Pearson correlation test: N = 204, r  =  0.56, *P* < 0.001) and we retained only the number of spiders per cell for further analyses. Habitat preference of spiders was based on the ecological characterisation of Central European spiders by Entling et al. [45]. They identified shading as a main niche dimension and derived niche positions on a gradient from open habitats to closed forest for 590 spider species. We considered spiders with niche position in the upper half of the shading gradient as forest-related species (Table 1). The two trap nests per site that were not used for prey assessment were collected in October 2010 to determine wasp abundance and parasitism. They were stored at 5°C until February 2011. Then, nesting tubes were singly transferred to test tubes and stored in a greenhouse for hatching of *T. figulus* and any parasitoids that have developed on them. Abundance of *T. figulus* can be measured as the number of colonized nesting tubes ( =  number of nest-building females) or as the number of brood cells that have been built ( =  productivity of nest-building females). In our study, both measures were strongly correlated (Pearson correlation test: N = 30, r  =  0.98, *P* < 0.001), such that we retained only the number of colonized nesting tubes as a measure of wasp abundance. We additionally measured the number of cells per occupied nesting tube as a measure of individual female investment in offspring production. Emerged parasitoids were determined as close as possible to the species level and parasitism rate was assessed by calculating the proportion of brood cells parasitized in each site. As additional measures of fitness, we assessed the sex-ratio and weight of emerged male and female offspring of *T. figulus* in 150 randomly chosen nesting tubes (50 per isolation level) with no mortality. Measures of weight were obtained for 610 males and 256 females.

**Table 1:** Spider species recorded in the brood cells of *Trypoxylon figulus*.

Family	Species	Individuals		Shading index^1^
		Adults	Immatures	
Araneidae	*Araneus angulatus* Clerck, 1757	0	2	na
	*Araneus diadematus* Clerck, 1757	0	1	0.38
	*Araneus* sp.	7	82	
	*Araniella cucurbitina* (Clerck, 1757)	47	0	0.44
	*Araniella opisthographa* (Kulczynski, 1905)	18	0	0.54
	*Araniella* sp.	17	88	
	*Argiope bruennichi* (Scopoli, 1772)	1	132	0.24
	*Araneus sturmi* (Hahn, 1831)	64	0	0.77
	*Mangora acalypha* (Walckenaer, 1802)	588	7	0.30
	*Nuctenea umbratica* (Clerck, 1757)	0	8	0.33
	undet.	4	11	
Linyphiidae	*Floronia bucculenta* (Clerck, 1757)	1	6	0.40
	*Linyphia hortensis* Sundevall, 1830	3	0	0.62
	*Linyphia triangularis* (Clerck, 1757)	21	65	0.53
	*Meioneta rurestris* (C.L.Koch, 1836)	8	0	0.25
	*Microlinyphia pusilla* (Sundevall, 1830)	7	3	0.27
	*Neriene radiata* (Walckenaer, 1842)	4	0	0.60
	*Tenuiphantes tenuis* (Blackwall, 1852)	1	0	0.31
	undet.	19	42	
Salticidae	*Heliophanus flavipes* (Hahn, 1832)	1	1	0.17
	undet.	1	0	
Tetragnathidae	*Metellina* sp.	1	39	>0.50
	*Tetragnatha extensa* (Linnaeus, 1758)	22	7	0.30
	undet.	2	27	
Theridiidae	*Anelosimus vittatus* (C.L.Koch, 1836)	1	0	0.28
	*Cryptachaea riparia* (Blackwall, 1834)	21	1	0.18
	*Enoplognatha ovata* (Clerck, 1757)	158	34	0.46
	*Enoplognatha* sp.	9	146	
	*Neottiura bimaculata* (Linnaeus, 1767)	2	0	0.26
	*Parasteatoda lunata* (Clerck, 1757)	1	0	0.18
	*Phylloneta impressum* L. Koch, 1881	3504	2263	0.26
	*Phylloneta sisyphia* (Clerck, 1757)	3	0	0.37
	*Platnickina tincta* (Walckenaer, 1802)	6	0	0.50
	*Theridion pinastri* L. Koch, 1872	24	1	na
	*Theridion varians* Hahn, 1833	13	0	0.31
	*Theridion* sp.	2	7	
	undet.	12	15	
Thomisidae	*Philodromus* cf. *aureolus* (Clerck, 1757)	1	1	na
	*Philodromus cespitum* (Walckenaer, 1802)	0	1	
	undet.	0	1	na
Undetermined		1	5	
Total		4595	2995	

1 Niche positions range from 0 for the species with maximal preference for open habitats to 1 for the species with maximal preference for closed forest [45] (See Materials and methods for details). na: not available.

### Abiotic variables

Local climate and altitude were measured at each site to test for possible confounding influences. Altitude was obtained from topographic maps to the nearest 5 m. Air temperature and humidity were recorded at each site once per hour with a data logger (Hygrochron™ iButtons) and the mean over the flying season (May-August 2010) was calculated and used in the analyses. Wind speed was recorded every fifteen seconds during three minutes with a cup vane anemometer (PCE-A420) and the values were averaged. The recording was repeated once per month at each site between May and September and the mean was used in the analyses.

### Statistical analyses

Generalized linear models were used to test the effects of landscape variables on 1) the abundance of *T. figulus* (number of occupied nesting tubes), 2) the number of brood cells per nesting tube, 3) the mean number of spiders per brood cell, 4) the proportion of forest-related spiders, 5) parasitism rate, and 6) offspring sex-ratio. Nominator and denominator degrees of freedom are reported with F-values for models containing habitat isolation and the proportion of woody habitat as explanatory variables. The denominator degrees of freedom vary between models because four sites (two connected and two isolated) contained less than five occupied nesting tubes and were only included in the modelling of the abundance of *T. figulus*. One additional site (connected) was removed from all spider-related models due to the low number of spiders collected. Quasi-binomial distribution was used on proportions (proportion of forest-related spiders, parasitism rate, and offspring sex-ratio), quasi-poisson distribution was used on counts (abundance of *T. figulus*), and Gaussian distribution was used for the remaining dependent variables. Generalized linear mixed models, with brood cell nested within site as a random factor and Gaussian distribution, were used to test the effects of landscape variables on the weight of male and female offspring. We controlled for possible confounding effects of four abiotic variables - temperature, humidity, wind and altitude - by entering each one at a time as first variable in the models and using type I sums of squares. We similarly controlled for any effect of tube diameter on the mean number of spiders per brood cell. Post-hoc tests (Tukey’s) were performed to test for significant differences between the levels of the explanatory variables. Possible competition for nesting tubes was assessed by performing a Pearson correlation test between the number of nesting tubes occupied by *T. figulus* and the number of nesting tubes occupied by other species. Model fit was evaluated by visual inspection of residual plots. All analyses were done with R version 2.14.1 [46].

Additionally, structural equation modelling (SEM, [47]) was used to test for direct and indirect effects of landscape structure on wasp abundance. A matrix of correlations was first calculated using Pearson’s correlation coefficients. This matrix served as a basis for SEM calculations. Monte Carlo resampling was used to account for the small sample size. We generated N = 1000 random data sets from normal variates with the observed means and variances and fitted them to our model. Results were then compared to our original data. The model fitted better in 171 bootstrap samples and fitted worse or failed to fit in 829 bootstrap samples. As a result, we did not reject the null hypothesis that the model is correct (*P*  =  0.829). The structural equation model and the resampling procedure were performed in AMOS 5.0.1 [48].

## Results

### Generalized linear models

**Figure 1:**
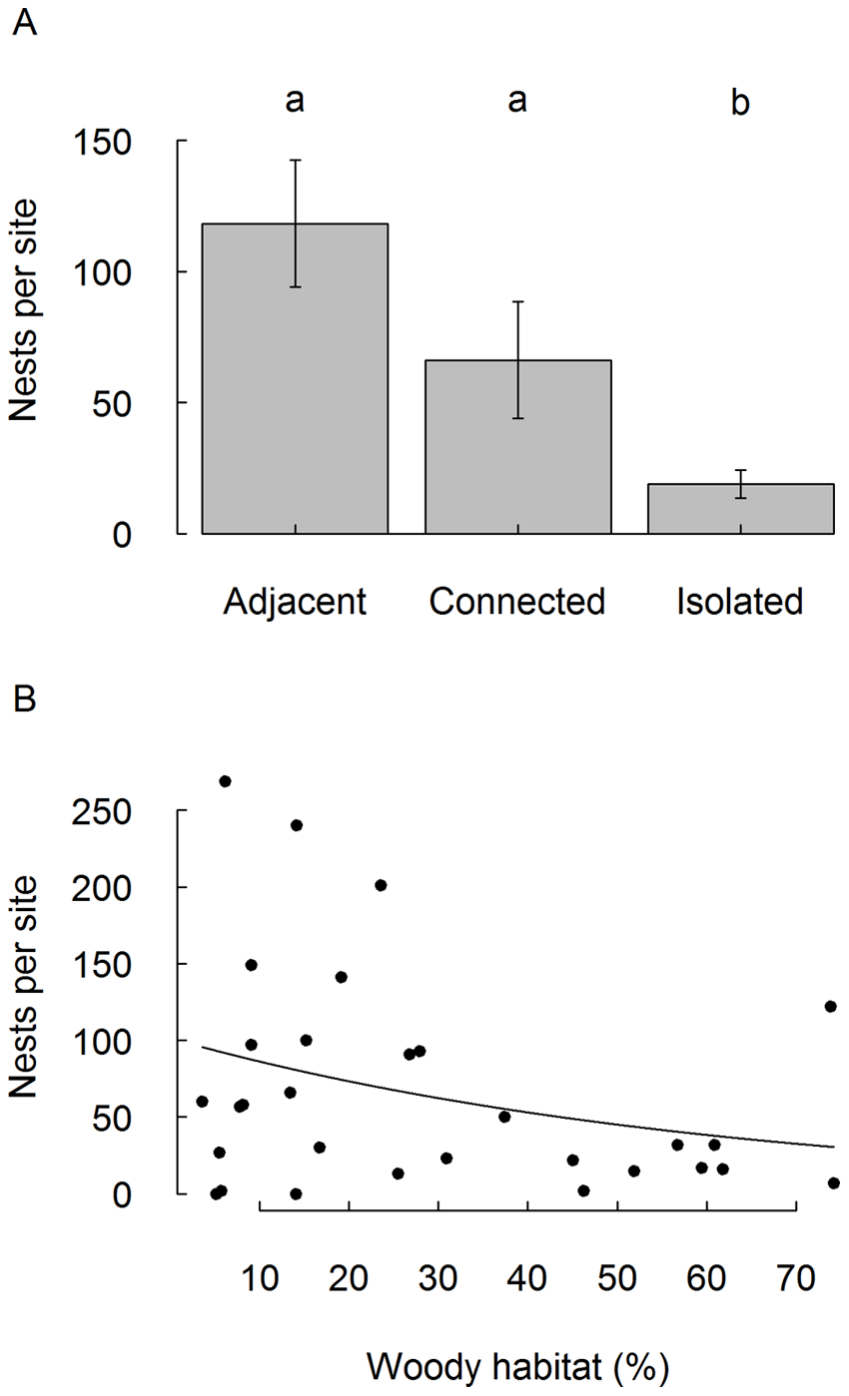
Abundance of *Trypoxylon figulus* in relation to landscape parameters. a) Local habitat isolation, b) Amount of woody habitat in the landscape (Poisson regression line: *y*  =  exp(4.62 −0.016*x*)). Error bars indicate standard error of the mean. Significant differences are represented by different letters (Tukey’s test, *P* ≤ 0.05). Nests refer to the mean number of occupied nesting tubes.

We collected 912 reed tubes, containing a total of 4483 brood cells of *T. figulus*. Abundance of *T. figulus* was highest in sites adjacent to forest and was significantly lower in isolated sites (F_2,26_  =  10.1, *P* < 0.01, Figure 1a). Additionally, wasp abundance significantly decreased with increasing proportion of woody habitat (F_1,26_  =  5.0, *P*  =  0.03, Figure 1b). The number of breeding cell per nesting tube was neither significantly related to isolation (F_2,22_  =  1.8, *P*  =  0.17) nor to the amount of woody habitat (F_1,22_  =  0.2, *P*  =  0.65). Regarding possible competition for nesting tubes, we found no negative relationship between the number of nesting tubes occupied by *T. figulus* and by other species (Pearson correlation test: N  =  30, r  =  0.05, *P*  =  0.79).

**Figure 2:**
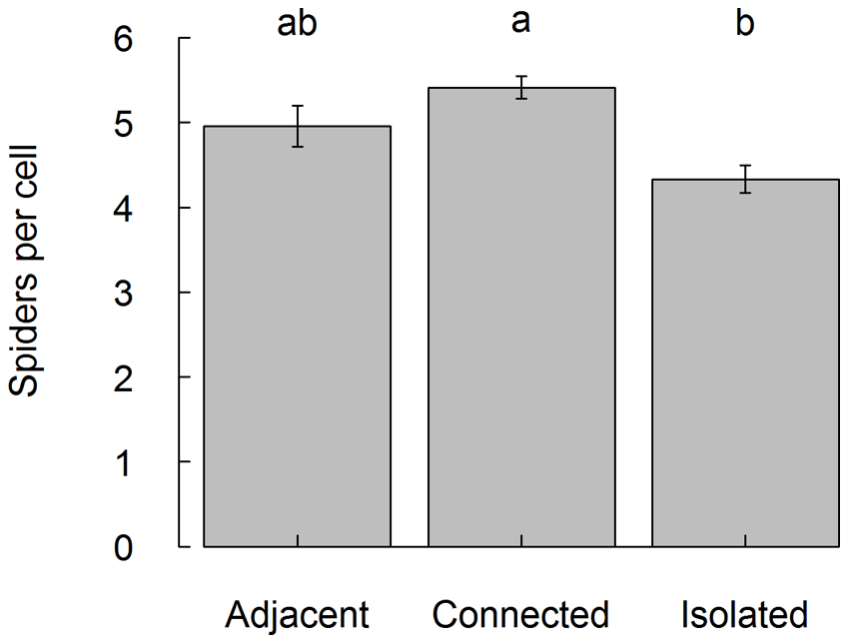
Number of spiders per single brood cell in relation to local habitat isolation. The mean number of spider individuals is significantly lower in isolated sites compared to connected sites. Error bars indicate standard error of the mean. Significant differences are represented by different letters (Tukey’s test, *P* ≤ 0.05).

7591 spiders were collected from 1496 intact brood cells. Each cell contained between 1 and 24 spider individuals (mean ± sd  =  5.1 ± 2.9). We identified 29 spider species from 6 families (Table 1). Theridiidae were dominant (82 %), especially the species *Phylloneta impressa*, which accounted for about 80 % of all spiders collected. The second most abundant family was Araneidae (14 %), in which *Mangora acalypha* was particularly numerous and accounted for 8 % of all spiders. Other spider families accounted for less than 3 % each. The cells of nests in isolated sites contained significantly less spider individuals compared to connected sites, even after controlling for the diameter size of the nesting tube (F_2,20_  =  5.8, *P*  =  0.01, Figure 2). The proportion of forest-related species was very low in all sites (max  =  12.7 %) and positively related to the amount of woody habitat in 500 m radius (F_1,21_  =  5.1, *P*  =  0.03). Sex-ratio of *T. figulus* offspring was neither significantly related to habitat isolation (F_2_,_146_  =  0.1, *P*  =  0.92) nor to the amount of woody habitat (F_1_,_146_  =  0.9, *P*  =  0.35). The mean weight of male (F_2_,_21_  =  10.0, *P*  =  0.03) , but not of female offspring (F_2_,_21_ =  0.4, *P*  =  0.69), was slightly lower in sites adjacent to forest compared to connected and isolated sites (adjacent: 24.5 ± 7.1 mg, connected: 26.6 ± 7.1 mg, isolated: 28.2 ± 7.2 mg).

*T. figulus* brood cells were parasitized by at least 13 different parasitoid species (Table 2). With respect to the number of destroyed brood cells, the most damaging parasitoid species were *Melittobia acasta* (62.3 %), *Trichodes alvearius* (11.3 %), *Trichrysis cyanea* (10.2 %), and *Anthrax* sp. (5.6 %). Parasitism rate was neither significantly related to habitat isolation (F_2_,_22_  =  0.4, *P*  =  0.70) nor to the amount of woody habitat within the landscape (F_1_,_22_  =  0.1, *P*  =  0.71). None of the four abiotic variables (temperature, humidity, altitude or wind) significantly explained variation in wasp abundance, brood provision, offspring weight, or parasitism (Statistical tables are provided in supporting information (Table S1)). The proportion of male offspring decreased with increasing temperature (F_1_,_145_  =  9.0, *P* < 0.01) and increased with altitude (F_1_,_145_  =  0.0, *P*  =  0.03).

**Table 2:** Parasitoid species emerged from the brood cells of *Trypoxylon figulus*.

Order	Family	Species	Number of attacked brood cells	Proportion of attacked brood cells
Coleoptera	Dermestidae	*Megatoma undata* (Linnaeus, 1758)	21	1.2
		*Trogoderma glabrum* (Herbst, 1783)	6	0.3
		undet.	54	3.0
	Cleridae	*Trichodes alvearius* (Fabricius, 1792)	203	11.3
Diptera	Bombyliidae	*Anthrax* sp	101	5.6
	Chrysidae	*Chrysis ignita* (Linnaeus, 1758)	3	0.2
		*Trichrysis cyanea* (Linnaeus, 1758)	184	10.2
		undet.	47	2.6
Hymenoptera	Chalcidoidea	*Dibrachys* sp.	39	2.2
		*Melittobia acasta* (Walker, 1839)	1123	62.3
	Gasteruptiidae	*Gasteruption assectator* (Linnaeus, 1758)	1	0.1
	Ichneumonidae	*Ephialtes manifestator* (Linnaeus, 1758)	2	0.1
		*Thrybius brevispina* (Thomson, 1896)	2	0.1
		undet.	5	0.3
Undetermined			12	0.7

### Structural equation modelling

**Figure 3:**
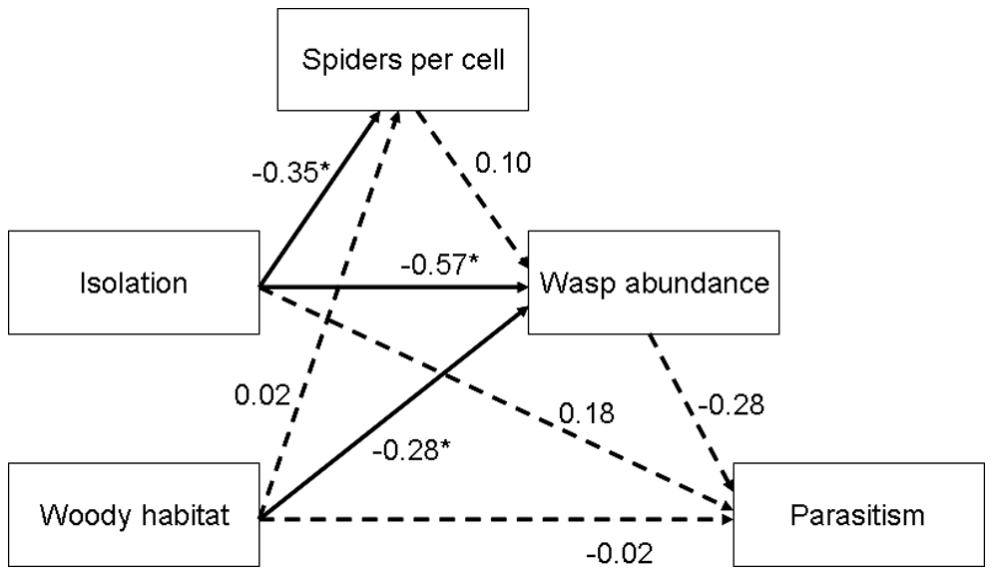
Structural equation modeling relating landscape parameters to the different trophic levels. The model shows all possible direct and indirect relationships between landscape parameters (local isolation and amount of woody habitat), prey abundance (spiders per cell), predator abundance (number of occupied nesting tubes), and predator parasitism rate. Standardized path coefficients are given next to the arrows (*P ≤ 0.05). Dotted arrows represent non-significant paths. Woody habitat: amount of woody habitat (%); Parasitism: proportion of parasitized brood cells.

The results from SEM (Figure 3) indicated that isolation significantly affected wasp abundance (estimate  =  − 48.6, S.E.  =  13.1, *P*  =  0.02) and the number of spiders per cell (estimate  =  −0.3, S.E.  =  0.1, *P*  =  0.05). However there was no significant direct effect of the number of spiders per cell on wasp abundance. In accordance to the GLM analysis, there was no relation between parasitism and either isolation or amount of woody habitat. In addition, SEM did not reveal any dependence of parasitism on host abundance.

## Discussion

*T. figulus* abundances were strongly reduced by habitat isolation, but unexpectedly decreased with increasing amount of woody habitat in the surrounding landscape (hypothesis (i)). This counterintuitive result confirms the importance of testing the independent effects of habitat amount and fragmentation *per se* [5].

### Prey abundance

Nests in isolated sites contained reduced number and total weight of spiders per brood cell and hence reduced food availability for the developing larvae (hypothesis (ii)). However, our results showed that the number of spiders per cell provides no significant explanation of wasp abundance. Besides prey abundance within the brood cells, the number of cells per nesting tubes, which indicates the number of offspring produced per female, may give indirect indication for limited food availability [49]. We found no difference in the number of brood cells per nesting tube between the different isolation levels or in relation to the amount of woody habitat. This suggests that *T. figulus* responds to prey abundance by limiting the number of provided spiders rather than the number of brood cells, or that food availability was not restricted enough to constrain the number of constructed cells. Reproductive success is not only determined by the number of produced offspring, but also by their quality. In several Hymenoptera species, larval food abundance determines the size and sex-ratio of the progeny [43], [44], [50], [51], [52]. Females breeding in habitats with poor food resources are therefore expected to produce lower quality and male-biased progeny. We tested this hypothesis and found no evidence for differences in sex-ratio or weight of female offspring among the different isolation levels. Unexpectedly, the mean weight of male offspring was lower in sites adjacent to forest than at isolated sites. Therefore, although we found evidence for reduced provisioning in isolated sites, this did not translate into reduced offspring weight. Overall, bottom-up effects of food resources do not appear to be a prime mechanism governing the abundance of *T. figulus* in our study area. The reasons for the low number of spiders per cell in isolated habitats could be related to spider abundance or accessibility in the field and remain to be studied.

### Prey spectrum

Like many other spider-hunting crabronid wasps, *T. figulus* hunted web-building spiders, whereas wandering spiders were hardly present in the larval provision [27], [28], [53]. In accordance with hypothesis (iii), wasps nesting adjacent to forest had significantly higher proportions of forest-related spiders in their diet. However, the generally very low proportions of wood-related spiders in the diet suggest that *T. figulus* preferably hunts in open habitats. Even though largely ubiquitous, the most hunted spider species, *P. impressa*, does not occur in closed forests [54], [55], [56]. This species is mainly associated with open landscapes [45], where it occasionally occurs on trees [57], [58]. Likewise, most other spider species found in the brood cells of *T. figulus* are common species of the open agricultural landscape [57], [59]. This strong relationship to open-landscape related spiders might explain why the abundance of *T. figulus* decreased with the amount of woody area in the landscape. Despite the large dominance of *P. impressa*, the presence of further species in the larval diet suggests that *T. figulus* is not restricted by the availability of a specific prey. Although opportunistic hunting is a common ecological feature within the genus *Trypoxylon* [27], [28], [29], some cases of specialisation have been reported [29], [60], and there is evidence that some predatory wasps demonstrate prey choice [49], [61]. To date, it is the first time that such a strong prevalence of a single species is found within the genus *Trypoxylon* and it would be interesting to further investigate the specific relationship between *T. figulus* and *P. impressa*. For example additional information may be gained by sampling spiders in the landscape and compare their frequency of occurrence with the frequency of occurrence in the brood cells. This could also reveal if the relationship between larval provisioning and actual food availability changes with habitat fragmentation [62]. The observed dominance of *P. impressa* was even more unexpected as Theridiidae are 3D-web building spiders, which are considered less attractive because their web architecture should be a good defensive structure against spider hunting wasps [63], [64], but see [53].

### Parasitism

We found no effect of habitat loss or isolation on parasitism rate. Therefore, our results did not conform to the expected enemy-release situation in isolated sites (hypothesis (iv)); [26], [65]. Because of their top position in the food chain, which often results in small and fluctuating population sizes, parasitoids are expected to be particularly affected by habitat fragmentation [24], [37], [66], [67]. However, fragmentation effects may be smaller for generalist parasitoids that occur in alternative preys [68]. Indeed, the most abundant parasitoid in our study (*Melittobia acasta*) has a very broad host spectrum including species occurring in various habitats [69], [70]. Beside the lack of any landscape effect on the parasitism rate of *T. figulus*, we did not find host density-dependence in parasitism rate. This outcome suggests that parasitism is not an effective determinant of fragmentation-related mortality and that *T. figulus* abundances were not regulated by higher trophic levels.

## Conclusion

We showed that population abundances of the wasp *T. figulus* decrease with habitat isolation and with the amount of forest in the landscape. The wasp diet was dominated by spiders typical of open agricultural land, but more wood-related spiders were collected at sites adjacent to forest. In addition, food provisioning to wasp larvae was lowest but weight of male offspring highest at isolated sites, while parasitism was unaffected by the landscape context. Overall, the studied trophic interactions provide no significant explanation for wasp abundance. Thus, further factors, such as dispersal and habitat preference are likely to play a role in the observed responses of *T. figulus* to habitat fragmentation.

## Supporting Information

Table S1 Results of sequential tests with potential confounding environmental variables introduced first in the models.
(DOCX)
