# Supplementary material for: Effects of Habitat Fragmentation on Abundance, Larval Food and Parasitism of a Spider-Hunting Wasp
Source: PLoS One. 2013 Mar 14;8(3):e59286. doi: 10.1371/journal.pone.0059286 (PMC3597609; doi:10.1371/journal.pone.0059286)
Supplement: Table S1 — Results of sequential tests with potential confounding environmental variables introduced first in the models. (DOCX) [file pone.0059286.s001.docx]

**Supporting information**

Table S1: Results of sequential tests with potential confounding environmental variables introduced first in the models.

| Response variables | | | | *T. figulus* abundance | | | |  |  | | Number of spiders pro cell | | | | |  |  | | Parasitism |  | |  |
| --- | --- | --- | --- | --- | --- | --- | --- | --- | --- | --- | --- | --- | --- | --- | --- | --- | --- | --- | --- | --- | --- | --- |
|  | df | | | | *F* | *p* | | |  | df | | | *F* | *p* | |  | df | | *F* | *p* | |  |
| Temperature | 1, 25 | | | | 1.87 | 0.183 | | |  | 1, 20 | | | 0.39 | 0.539 | |  | 1, 21 | | 0.06 | 0.814 | |  |
| Isolation | 2, 25 | | | | 10.64 | < 0.001 | | |  | 2, 20 | | | 5.47 | 0.013 | |  | 2, 21 | | 0.31 | 0.734 | |  |
| Woody habitat | 1, 25 | | | | 8.71 | 0.007 | | |  | 1, 20 | | | 0.54 | 0.473 | |  | 1, 21 | | 0.12 | 0.728 | |  |
| Humidity | 1, 25 | | | | 0.00 | 0.982 | | |  | 1, 20 | | | 0.08 | 0.777 | |  | 1, 21 | | 1.94 | 0.178 | |  |
| Isolation | 2, 25 | | | | 10.26 | 0.001 | | |  | 2, 20 | | | 5.61 | 0.012 | |  | 2, 21 | | 0.07 | 0.937 | |  |
| Woody habitat | 1, 25 | | | | 5.23 | 0.031 | | |  | 1, 20 | | | 0.43 | 0.518 | |  | 1, 21 | | 0.39 | 0.541 | |  |
| Altitude | 1, 25 | | | | 0.00 | 0.982 | | |  | 1, 20 | | | 0.10 | 0.756 | |  | 1, 21 | | 0.15 | 0.704 | |  |
| Isolation | 2, 25 | | | | 10.26 | 0.001 | | |  | 2, 20 | | | 5.67 | 0.011 | |  | 2, 21 | | 0.34 | 0.716 | |  |
| Woody habitat | 1, 25 | | | | 5.23 | 0.031 | | |  | 1, 20 | | | 0.55 | 0.466 | |  | 1, 21 | | 0.06 | 0.810 | |  |
| Wind | 1, 25 | | | | 3.05 | 0.093 | | |  | 1, 20 | | | 0.33 | 0.571 | |  | 1, 21 | | 0.77 | 0.391 | |  |
| Isolation | 2, 25 | | | | 9.03 | 0.001 | | |  | 2, 20 | | | 5.50 | 0.013 | |  | 2, 21 | | 0.60 | 0.557 | |  |
| Woody habitat | 1, 25 | | | | 4.97 | 0.035 | | |  | 1, 20 | | | 0.54 | 0.470 | |  | 1, 21 | | 0.00 | 0.973 | |  |
|  |  | | | |  |  | | |  |  | | |  |  | |  |  | |  |  | |  |
|  |  | | | |  |  | | |  |  | | |  |  | |  |  | |  |  | |  |
| Response variables | |  | Male weight | | | |  | |  | | | Female weight | | |  |  |  | Offspring sex-ratio | | |  |  |
|  | df | | | | *F* | *p* | | |  | df | | | *F* | *p* | |  | df | | *F* | *p* | |  |
| Temperature | 1, 20 | | | | 1.01 | 0.327 | | |  | 1, 20 | | | 0.07 | 0.793 | |  | 1, 145 | | 9.00 | < 0.01 | |  |
| Isolation | 2, 20 | | | | 3.50 | 0.050 | | |  | 2, 20 | | | 0.39 | 0.685 | |  | 2, 145 | | 1.07 | 0.35 | |  |
| Woody habitat | 1, 20 | | | | 1.27 | 0.273 | | |  | 1, 20 | | | 0.01 | 0.940 | |  | 1, 145 | | 0.16 | 0.69 | |  |
| Humidity | 1, 20 | | | | 3.50 | 0.074 | | |  | 1, 20 | | | 2.48 | 0.132 | |  | 1, 145 | | 2.74 | 0.1 | |  |
| Isolation | 2, 20 | | | | 3.98 | 0.035 | | |  | 2, 20 | | | 0.31 | 0.737 | |  | 2, 145 | | 0.60 | 0.55 | |  |
| Woody habitat | 1, 20 | | | | 0.87 | 0.361 | | |  | 1, 20 | | | 0.04 | 0.847 | |  | 1, 145 | | 0.39 | 0.53 | |  |
| Altitude | 1, 20 | | | | 0.00 | 0.965 | | |  | 1, 20 | | | 0.35 | 0.560 | |  | 1, 145 | | 4.84 | 0.03 | |  |
| Isolation | 2, 20 | | | | 3.89 | 0.038 | | |  | 2, 20 | | | 0.36 | 0.701 | |  | 2, 145 | | 0.04 | 0.96 | |  |
| Woody habitat | 1, 20 | | | | 1.67 | 0.202 | | |  | 1, 20 | | | 0.03 | 0.871 | |  | 1, 145 | | 0.02 | 0.89 | |  |
| Wind | 1, 20 | | | | 0.58 | 0.455 | | |  | 1, 20 | | | 0.10 | 0.760 | |  | 1, 145 | | 0.10 | 0.75 | |  |
| Isolation | 2, 20 | | | | 4.18 | 0.030 | | |  | 2, 20 | | | 0.48 | 0.624 | |  | 2, 145 | | 0.19 | 0.83 | |  |
| Woody habitat | 1, 20 | | | | 2.65 | 0.119 | | |  | 1, 20 | | | 0.06 | 0.815 | |  | 1, 145 | | 0.93 | 0.33 | |  |
|  |  | | | |  |  | | |  |  | | |  |  | |  |  | |  |  | |  |
